# Android Robot Promotes Disclosure of Negative Narratives by Individuals With Autism Spectrum Disorders

**DOI:** 10.3389/fpsyt.2022.899664

**Published:** 2022-06-15

**Authors:** Hirokazu Kumazaki, Taro Muramatsu, Yuichiro Yoshikawa, Yoshio Matsumoto, Keiji Takata, Hiroshi Ishiguro, Masaru Mimura

**Affiliations:** ^1^Department of Neuropsychiatry, Unit of Translational Medicine, Nagasaki University Graduate School of Biomedical Sciences, Nagasaki, Japan; ^2^Department of Preventive Intervention for Psychiatric Disorders, National Center of Neurology and Psychiatry, National Institute of Mental Health, Tokyo, Japan; ^3^College of Science and Engineering, Kanazawa University, Kanazawa, Japan; ^4^Department of Neuropsychiatry, Keio University School of Medicine, Tokyo, Japan; ^5^Human Augmentation Research Center, National Institute of Advanced Industrial Science and Technology, Chiba, Japan; ^6^Department of Systems Innovation, Graduate School of Engineering Science, Osaka University, Osaka, Japan; ^7^Research Center for Child Mental Development, Kanazawa University, Kanazawa, Japan

**Keywords:** self-disclosure, narrative, autism spectrum disorders, android robot, sentence completion test (SCT)

## Abstract

Many individuals with autism spectrum disorders (ASD) demonstrate some challenges with personal narrative writing. Sentence completion tests (SCT) is a class of semi-structured projective techniques and encourage respondents to disclose their private narratives. Even in SCT, only providing beginning of sentences is inadequate to compensate atypicalities in their creativity and imagination, and self-disclosure is difficult for many individuals with ASD. It is reported that many individuals with ASD often achieve a higher degree of task engagement through interactions with robots and that robotic systems may be useful in eliciting and promoting social communication such as self-disclosure for some individuals with ASD. There is a possibility that exemplification by android robots in place of human interviewers can result in a higher degree of task engagement for individuals with ASD. The objective of this study was to investigate whether additional exemplifications by android robots in the SCT can prompt self-disclosure for individuals with ASD. We compared the difference in disclosure statements and subjective emotion in the testing paper of the SCT in additional exemplification by an android robot and a human interviewer. In addition, we assessed the disclosure statements and subjective emotions in the SCT, for which exemplifications were written on testing paper to make the comparison. Our quantitative data suggested that exemplification by android robot promoted more self-disclosure, especially about the negative topic compared to exemplification by a human interviewer and that written on test paper. In addition, the level of participant embarrassment in response to exemplification by the android robot seemed to be lower compared to that in the human interviewer condition. In the assessment and support for individuals with ASD, eliciting self-disclosure is a pressing issue. It is hoped that the appropriate use of robots will lead to a better understanding and support for their application.

## Introduction

Self-disclosure is the process by which people reveal personal information about themselves to others and is important in all types and stages of social relationships (1). Individuals with autism spectrum disorders (ASD) display impairments in social communication and interaction, often including challenges related to appropriate engagement in conversation. They sometimes hesitate to disclose information to others due to challenges in understanding such interchanges or recognizing the potential value in relational reciprocity, as well as differences in social motivation (2). Previous studies have consistently demonstrated that individuals with ASD provide fewer and shorter self-disclosure statements of personal narratives than individuals with typical development (2–5).

Sentence completion test (SCT) (6–8) is a class of semi-structured projective techniques. SCT typically provide respondents with beginnings of sentences, referred to as “stems,” and respondents then complete the sentences in ways that are meaningful to them. In general, directly answering an examiner tends to make the individual self-conscious and is likely to make him or her nervous. However, while administering the SCT, when an individual is advised to write on testing paper the first idea that occurs to him or her, he or she yields important information regarding various areas of an individual's life because it is easy to administer. Therefore, with such an advantage, the SCT encourages the respondents to disclose their private narratives. The responses are believed to provide indications of attitudes, beliefs, motivations, or other mental states (9).

Individuals with ASD tend to show impoverished creativity as well as deficient imagination (10). Previous studies suggest that individuals with ASD demonstrate some challenges with personal narrative writing compared to the ability of individuals in the control group (11–13). Even in SCT, only providing beginning of sentences is inadequate to compensate atypicalities in their creativity and imagination, and self-disclosure is difficult for many individuals with ASD. Exemplifications by human interviewers have the potential to compensate for creativity and imagination, which is expected to be useful for prompting self-disclosure. However, many individuals with ASD cannot easily sustain high motivation and concentration needed for dialog with humans (14, 15). Intensive sensory processing may be affected by the dynamic facial features and expressions of human beings, which are likely to induce sensory and emotional overstimulation and distractions and are linked to worsening social anxiety (16). This can, consequently, interfere with their thinking and answering, as these individuals tend to actively avoid sensory stimulations and instead focus on more predictable elementary features.

Individuals with ASD often achieve a higher degree of task engagement through interactions with robots than through interactions with humans (17–24). Robots allow them to control and replicate a scene with smooth and accurate information presentation, contributing to a more structured and standardized intervention. Unlike human beings, robots that operate within predictable and lawful systems provide a highly structured learning environment that help individuals with ASD to focus on relevant stimuli.

An android robot is a humanoid robot with an appearance and movements resembling those of an actual human. It can exhibit facial expressions (e.g., smiling, nodding, and brow movements) during speech and provide subtle non-verbal cues. It can potentially simulate actions that are performed in daily life to some extent. It is very similar to humans but, since it is a robot, it is still much simpler than humans. There is increasing anecdotal evidence for the fact that individuals with ASD may have unique opportunities to use android robots to assist them. A previous study reported a novel intervention using android robots to reduce social anxiety (25, 26). It has been reported that many of individuals with ASD can easily sustain high motivation and concentration for dialog with android robots and that robotic systems may be useful in eliciting and promoting aspects of social communication, such as self-disclosure, in some individuals with ASD (5). Therefore, there is a possibility that exemplification by android robots in place of human interviewers can result in a higher degree of task engagement for individuals with ASD by reducing their social anxiety and helping their imagination and creativity; thus, interactions with android robots are linked to promoting self-disclosure by these individuals.

The objective of this study was to investigate whether additional exemplifications by android robots in the SCT can prompt self-disclosure for individuals with ASD. We compared the difference in disclosure statements and subjective emotion in the testing paper of the SCT in additional exemplification by an android robot and a human interviewer. In addition, we assessed the disclosure statements and subjective emotions in the SCT, for which exemplifications were written on testing paper to make the comparison. We also investigated the relationship between individual characteristics and self-disclosure statements. A greater understanding of the relationship could provide insight into developing a future therapeutic android robot to manage self-disclosure difficulties for individuals with ASD.

## Materials and Methods

### Participants

The present study was approved by the ethics committee of Kanazawa University. Participants were recruited by flyers that explained the content of the experiment. All procedures involving human participants were conducted in accordance with the ethical standards of the institutional and/or national research committee and the 1964 Declaration of Helsinki and its later amendments or comparable ethical standards. After receiving a complete explanation of the study, all participants and their guardians agreed to participate. Written informed consent was obtained from participants and/or from minor participants' legal guardian for the publication of any potentially identifiable images or data included in this article. The inclusion criteria included (1) having a diagnosis of ASD based on the Diagnostic and Statistical Manual of Mental Disorders, Fifth Edition (DSM-5) (27) from the supervising study psychiatrist, (2) having an IQ ≥70, and (3) not taking medication. The exclusion criteria for the ASD group were medical conditions associated with ASD (e.g., fragile X mental retardation 1, Rett syndrome, and Shank3). To exclude other psychiatric diagnoses, the Mini-International Neuropsychiatric Interview (28) was administered. At the time of enrollment, the diagnoses of all participants were confirmed by a psychiatrist with more than 15 years of experience in ASD using the criteria in the DSM-5 and standardized criteria taken from the Diagnostic Interview for Social and Communication Disorders (DISCO) (29). The DISCO has been reported to have good psychometric properties (30).

All participants completed the Autism Spectrum Quotient-Japanese version (AQ-J) (31), which has been used in the evaluation of ASD-specific behaviors and symptoms. The AQ-J is a short questionnaire with five subscales (social skills, attention switching, attention to detail, imagination, and communication). Previous work with the AQ-J has been replicated across cultures (32) and ages (33, 34). Notably, the AQ is sensitive to the broader autism phenotype (35).

Full-scale IQ scores were measured by either the Wechsler Intelligence Scale for Children—Fourth Edition, the Wechsler Adult Intelligence Scale–Third Edition or the Japanese Adult Reading Test (JART) (36). The latter is a standardized cognitive function test to estimate the premorbid IQ of examinees with cognitive impairments. The JART has good validity for measuring IQ compared to that measured using the WAIS-III. The JART results can be compared to those of the WAIS-III (36).

The participants' severity of social anxiety symptoms was measured using the Liebowitz Social Anxiety Scale (LSAS) (37). This clinician-administered scale consists of 24 items, including 13 items that describe performance situations and 11 items that describe social interaction situations. Each item was separately rated for “fear” and “avoidance” using a 4-point categorical scale. According to receiver operating curve analyses, an LSAS score of 30 is correlated with minimal symptoms and is the best cutoff value for distinguishing individuals with and without social anxiety disorder (38).

The Adolescent/Adult Sensory Profile [AASP (39)] is a self-report questionnaire measuring sensory processing in individuals aged 11 years and older. The internal consistency coefficients of the AASP range from 0.64 to 0.78 for the quadrant scores. In this study, before the experiment, the participants indicated how often they exhibited certain behaviors related to sensory experiences using a scale of 1–5 indicating “almost never” (score of 1) to “almost always” (score of 5). The AASP examines four different “quadrants” of sensory processing: low registration, sensation seeking, sensory sensitivity, and sensation avoiding. As the AASP does not categorize responses according to individual “perceptual domains” (such as auditory, visual, tactile, etc.), a perceptual domain analysis was not performed in this study.

### Robotic System

**Figure 1:**
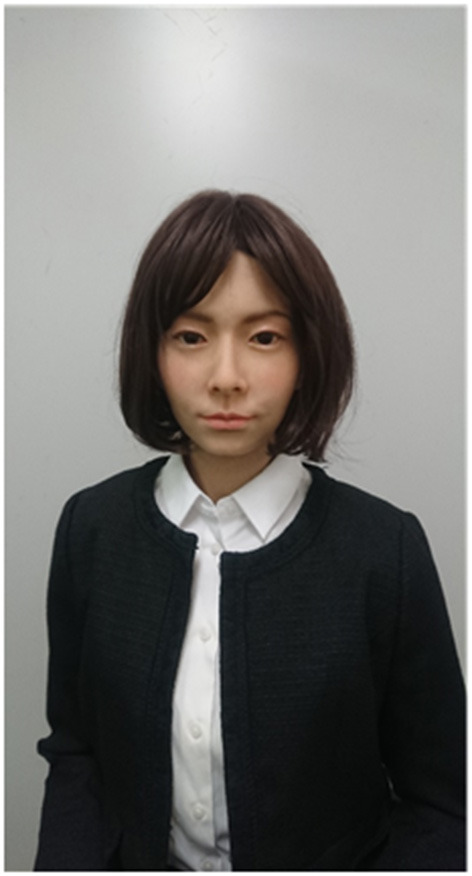
A-Lab Android ST.

The android robot used in this study was A-Lab android ST (Figure 1) (A-Lab Co., Ltd. Chiyoda-ku, Tokyo, Japan.), which is a female humanoid robot with an appearance similar to that of a real person. Its artificial body has the same proportions, facial features, hair color, and hairstyle as a human. The synthesized voice of the android robot is also similar to that of an actual person. To elicit the belief that the robots behaved and responded autonomously without fail, we adopted a remote-control system similar to that conventionally used in robotics studies (40). The android robot incorporated changes in facial expression (i.e., smiling, nodding, and brow movements) during speech.

### Procedure

**Figure 2:**
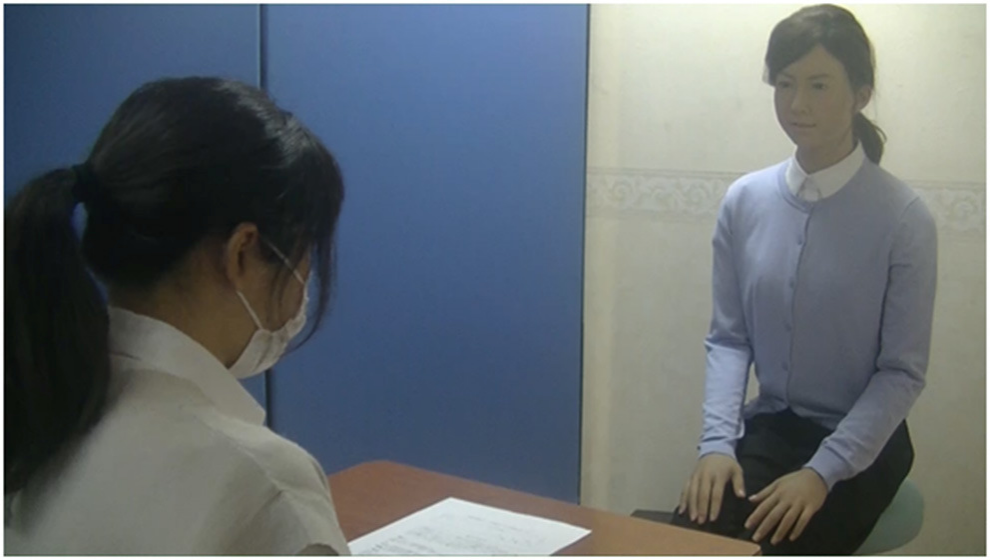
An example of how an android robot provides exemplification. The researcher operated the android robot from a different room. When the researcher pushed a button, the robot began to speak exemplification according to previously prepared scripts. Participants then filled out their private narratives on the testing paper of SCT. Right: android robot; Left: participant.

We used the first 30 questions in the Japanese version of SCT called the SEIKEN SCT (Sano & Makita, 1960). In this study, unlike the standard SCT, which presents the beginning of sentences, additional short exemplifications were presented. The procedure for making short exemplifications was as follows. First, two psychologists chose sample responses from the SCT, which are listed in the manual. Second, they drafted short exemplifications while referencing sample responses independently. Then, they revised the draft so that individuals with ASD whose IQ was above 70 could understand the meaning. Finally, we confirmed that they can understand the meaning of short examples in our preliminary experiment. Please refer to Supplementary Material 1 for the content of the exemplifications and beginnings of the sentences. We designed three conditions, exemplification by android robot, by a human interviewer, and in a written passage on testing paper. Each participant experienced 10 questions in each of the three conditions in random order, all of which were guided and took place in a standard clinical assessment room. The conditions were performed in different rooms. We ensured a balance in the order of conditions to a certain extent. Please refer to Supplementary Material 2 for this order. Figure 2 provides an example of how an android robot provided the exemplification. The person in Figure 2 has given written and informed consent for publication of this image. The android robot was operated by the researcher seated in a different room. When a button was pushed by the researcher, the android robot began to speak according to previously prepared scripts. The operator could monitor participants' answers via video. The human interviewer was a 25-year-old Japanese woman. Each trial ended when the participant answered all questions or communicated that he or she did not wish to answer the question.

At the close of the session, participants completed a self-report survey using a 7-point Likert scale to rank their level of stress, embarrassment, boredom, and enjoyment in each session. Two professional psychologists counted the number of words indicating self-disclosure that were written on the testing paper in each condition. The psychologists also assessed the number of topics about positive and negative self-disclosure. Positive self-disclosure was defined as the expression of positive narratives in the past, present, and future. Negative self-disclosure was defined as the expression of negative narratives in the past, present, and future. In addition, the two professional psychologists evaluated the items of positive and negative self-disclosures separately, and items that were rated in agreement by both psychologists were adopted.

### Data Analysis

We performed statistical analysis using SPSS version 24.0 (IBM, Armonk, NY, USA). Differences in the number of words and topics about positive and negative disclosure written on the testing paper of SCT as well as self-report ratings between exemplification by android robot vs. human interviewer and exemplification by android robot vs. written passages on testing paper were analyzed using the Wilcoxon signed-rank test. Spearman's rank correlation coefficients were used to explore the relationships among demographic data (i.e., age, full-scale IQ, AQ-J score, LSAS score, and AASP subscore), the number of words, and number of topics related to positive and negative self-disclosure written on testing paper in the case of exemplification by android robot, by a human interviewer, and in a written passage on testing paper. An alpha level of 0.05 was used for these analyses.

## Results

In total, 21 individuals with ASD took part in the study. The details are presented in Table 1. All the participants completed the experimental procedure and the questionnaires.

**Table 1:** Descriptive statistics of the participants.

**Characteristics**	***n* = 21** **M (SD)**
Age (years)	21.7 (5.1)
Sex (Male: Female)	18:3
Full scale IQ	94.2 (12.0)
AQ-J	27.3 (6.5)
LSAS-J	59.3 (30.3)
**AASP**	
Low registration	38.3 (11.2)
Sensation seeking	36.9 (11.7)
Sensory sensitivity	38.2 (12.9)
Sensation avoiding	41.9 (11.9)

*M, mean; SD, standard deviation*.

We performed multiple comparison corrections for the number of words written on the testing paper of the SCT, the number of topics signifying positive disclosure, and the number of topics signifying negative disclosure using the Bonferroni method. The significance level after correction by the Bonferroni method was 0.025 each. There were no significant differences in the number of words written on the testing paper of the SCT between the conditions of exemplification by the android robot vs. the human interviewer (*p* = 0.443) and between the conditions of exemplification by the android robot vs. in a written passage on testing paper (*p* = 0.663). Significant trends were observed between exemplification by the android robot vs. the human interviewer in the number of topics signifying positive disclosure (*p* = 0.030). There were no significant differences in the number of topics signifying positive disclosure between exemplification by the android robot vs. in a written passage on paper (*p* = 0.218). Significant differences were observed between exemplification by the android robot vs. the human interviewer in the number of topics signifying negative disclosure (*p* = 0.020). Significant differences were also observed between exemplification by the android robot vs. in a written passage on paper in the number of topics signifying negative disclosure (*p* = 0.021). The details are presented in Table 2.

**Table 2:** The number of words and topics indicating positive and negative disclosure written on the SCT paper in response to exemplification by an android robot, a human interviewer, and a written passage on the test paper.

	**Exemplification by** **an android robot** **a M (SEM)**	**Exemplification by a human interviewer** **M (SEM)**	**Exemplification in a written passage on test paper** **M (SEM)**
The number of words	202.48 (22.50)	194.76 (20.01)	195.10 (20.98)
Topics indicating positive disclosure	2.48 (0.39)	1.57 (0.21)^b^	1.90 (0.30)
Topics indicating negative disclosure	1.67 (0.37)	0.90 (0.27)^a^	0.95 (0.24)^a^

*M, mean; SEM, standard error of the mean. ^a^statistically significant (p < 0.025) compared with android robot condition after adjustment for multiple tests using the Bonferroni method. ^b^ statistically significant (p < 0.05) compared with android robot condition after adjustment for multiple tests using the Bonferroni method*.

We performed multiple comparison corrections for the Likert scale to rank the participants' level of stress, embarrassment, boredom, enjoyment using the Bonferroni method. The significance level after correction by the Bonferroni method was 0.025 each. In the self-report survey to rank their level of embarrassment on a 7-point Likert scale, significant differences were observed between exemplification by the android robot and the human interviewer (*p* = 0.023). There were no significant differences in the Likert scale to rank the participants' level of embarrassment between exemplification by the android robot vs. in a written passage on the test paper of SCT (*p* = 0.064). There were no significant differences in the Likert scale in their rankings of stress level (*p* = 0.576), boredom (*p* = 0.888), and enjoyment (*p* = 0.820) between exemplification by the android robot vs. the human interviewer. There were no significant differences in the Likert scale in the rankings of their level of stress (*p* = 0.518), boredom (*p* = 0.781), and enjoyment (*p* = 0.268) between exemplification by the android robot vs. in a written passage on the test paper. The details are presented in Table 3.

**Table 3:** Overall impressions of each condition.

	**Exemplification by** **an android robot** **M (SEM)**	**Exemplification by a human interviewer** **M (SEM)**	**Exemplification in a written passage on test paper** **M (SEM)**
Stress	3.43 (0.39)	3.52 (0.44)	3.62 (0.46)
Embarrassment	2.00 (0.32)	2.86 (0.44)^a^	2.67 (0.45)
Boredom	3.05 (0.41)	3.10 (0.46)	3.14 (0.45)
Enjoyment	4.24 (0.37)	4.33 (0.39)	3.76 (0.43)

*M, mean; SEM, standard error of the mean. ^a^ statistically significant (p < 0.025) compared with android robot condition after adjustment for multiple tests using the Bonferroni method*.

In Spearman's rank correlation coefficients, there were no significant relationships between the number of topics about positive disclosure for exemplification by android robot and AQ score (*r* = −0.189, *p* = 0.413) and full IQ (*r* = 0.093, *p* = 0.689). We found a positive relationship between the number of topics signifying negative disclosure for exemplification by android robot and AQ score (*r* = 0.495, *p* = 0.023) and full IQ (*r* = 0.528, *p* = 0.014). There were no significant relationships between the number of topics signifying positive disclosure for exemplification by human interviewer and AQ score (*r* = −0.324, *p* = 0.152), between the number of positive disclosure topics and full IQ (*r* = 0.105, *p* = 0.650), between exemplification provided in a written passage on test paper and AQ score (*r* = −0.230, *p* = 0.315), between exemplification in a written passage on test paper and full IQ (*r* = 0.140, *p* = 0.544). There were also no significant relationships between the number of topics of negative disclosure for exemplification by human interviewer and AQ score (*r* = 0.354, *p* = 0.115), between the number of negative disclosure topics and full IQ (*r* = 0.421, *p* = 0.058), between exemplification provided in a written passage on test paper and AQ (*r* = 0.159, *p* = 0.490), between exemplification provided in a written passage on test paper and full IQ (*r* = 0.314, *p* = 0.166).

## Discussion

In this study, we compared the differences in disclosure statements and subjective emotions in SCT papers in exemplification by android robots and human interviewers. In addition, we assessed the disclosure statements and subjective emotions of participants performing the SCT, for which exemplifications were written on test paper to make the comparison. Our quantitative data suggested that exemplification by android robot promoted more self-disclosure, especially about the negative topic compared to exemplification by a human interviewer and that written on test paper. In addition, the level of participant embarrassment in response to exemplification by the android robot seemed to be lower compared to that in the human interviewer condition. While our sample sizes were somewhat small for statistical comparisons, our quantitative data indicated the usefulness of exemplifications by an android robot.

According to “The Social Motivation Theory of Autism” (41), individuals with ASD can be construed as extreme cases of diminished social motivation. Social motivation is a powerful force guiding human behavior. It can be described as a set of psychological dispositions and biological mechanisms biasing individuals to preferentially orient to the social world, seek and take pleasure in social interactions, and work to foster and maintain social bonds. Social motivation enables individuals with ASD to foster smooth relationships and promote coordination. Social communication intervention approaches are effective when individuals with ASD involve motivating activities and settings (15). Unlike human beings, humanoid robots operate within predictable and lawful systems and thus offer them a highly structured learning environment that can help them focus on relevant stimuli. Individuals with ASD have a higher degree of task engagement while communicating with humanoid robots than with human trainees and exhibit social behaviors toward humanoid robots (19).

When designing objects for use by individuals with ASD, researchers often subscribe to the notion that “simpler is better,” i.e., they will gravitate toward simple, mechanical objects (22, 42–45). Although android robots are very similar to humans, but still much simpler than humans. Above all, the high-technology behind the android robot may be favored by individuals with ASD. Moreover, a previous study reported that individuals with lower empathy (corresponding to a higher AQ) did not prefer “Baby Schema” (46). Given these backgrounds, it is natural that android robot is more effective than humans at interacting with them.

Exemplification by an android robot promotes self-disclosure, especially about negative topics, compared to exemplification by a human interviewer and that written on test paper. In this study, the level of embarrassment in response to exemplification by the android robot seemed to be lower compared to that elicited in the human interviewer condition. This outcome may be linked to an increase in self-disclosure about negative topics in the android setting.

We found a positive relationship between the number of topics indicating negative disclosure in response to exemplification by an android robot and AQ score and between the number of topics indicating negative disclosure in response to exemplification by an android robot and full IQ. A previous study (47) suggested that individuals with higher AQ scores preferring more android robots and that a certain level of intelligence is necessary to have a preference for these robots, which may explain the results of this study. In clinical settings, promoting negative self-disclosure is especially important. The results of this study suggest that the android system could be useful for eliciting negative self-disclosure, especially for individuals with ASD who have higher AQ and IQ scores.

The results of this preliminary efficacy study demonstrated that first exposure to android robot-mediated exemplification procedures promoted more self-disclosure about a negative topic. While the current study was not able to test habituation effects in any way, it represents one of the first systematic investigations in self-disclosure using an android robot for individuals with ASD. In future work, it would be important to evaluate habituation effects with android robots by observing interactions over an extended period of time.

In this study, we used an android robot that resembled a female human. The perception of the robot and gender identity of an individual with ASD may be considered when a robot is used for targeted, individualized social therapy strategies aimed at facilitating interactions and maximizing beneficial effects, considering that typically developed adults rate a robot of the opposite sex as more trustworthy, engaging, and credible than a robot matching the sex of the individual (48). Whether this finding is relevant to individuals with ASD has not been clarified. Moreover, using an android robot that considers participants' gender preference is needed to enhance the potential abilities of an android robotic intervention.

For successful robotic intervention, other viewpoints aside from selecting the optimal robot may be important. A previous study (49) suggested that the same robot is used, and the clothes and hairstyle of the robot across different contexts and roles need to be chosen. In addition, the authors suggested the need to change the robot's voice, length of sentences, and talking speed. Other than these factors, real-time response seems to be an important factor for the success of a robotic intervention. Considering these factors, we could take advantage of the potential abilities of an android robotic intervention to the fullest extent.

We would like to acknowledge several limitations of our study. First, our sample size was relatively small. Larger sample sizes are necessary to provide more meaningful data. In addition, most of our participants were male. Future research should include more female participants. Second, our interview was relatively short (i.e., participants experienced 10 questions in each condition, and the interviews took ~15 min); however, we judged that 15 min per session would be appropriate to meet the specific needs of individuals with ASD. In addition, all our participants were able to complete the trial. Third, we only included individuals with ASD. To elucidate the relationship between exemplifications by android robots and self-disclosure more clearly, it is important to study individuals without ASD and compare their data with those of individuals with ASD. At the time of this experiment, the Japanese government declared a state of emergency due to the spread of COVID-19, so we could not ask controls to participate in the experiment. Given that eliciting self-disclosure is a pressing issue for individuals with ASD, we had to conduct pilot studies even without controls. Additionally, their level of social anxiety is high. Although the level of social anxiety among individuals with ASD without intellectual disability is estimated to be higher than that of individuals with typical development (50), and social anxiety is common among individuals with ASD (51), future studies targeting individuals with ASD who have lower level of social anxiety are needed. Fourth, the characteristics of the human interviewer may certainly influence the quality and quantity of participants' self-disclosure. Our aim was to involve human interviewers who were matched according to the age and sex of the android (young adults and females). Therefore, we enlisted research assistants working in our laboratory (Japanese, female, average age: 25 years). Further investigation regarding the characteristics of the human interviewer (age, sex, and disposition) might yield interesting results. Fifth, in this study, we used android robot to provide the exemplification. However, it costs too high to prepare android robot. Future studies to investigate the possibility of using other robots are needed. Finally, it is possible that the within-subject design can be prone to the “carryover effect,” which may have affected the results.

Despite limitations, all participants were able to complete the study procedures, and the results suggest the usefulness of exemplification by android robots to promote self-disclosure for individuals with ASD. In the assessment and support for individuals with ASD, eliciting self-disclosure is a pressing issue. It is hoped that the appropriate use of robots will lead to a better understanding and support for their application.

## Data Availability Statement

The raw data supporting the conclusions of this article will be made available by the authors, without undue reservation.

## Ethics Statement

The studies involving human participants were reviewed and approved by Ethical committee, Kanazawa University. Written informed consent to participate in this study was provided by the participants' legal guardian/next of kin.

## Author Contributions

HK designed the study, conducted the experiment, carried out the statistical analyses, analyzed and interpreted the data, and drafted the manuscript. TM, YY, YM, KT, HI, and MM conceived of the study, participated in its design, assisted with data collection and the scoring of behavioral measures, analyzed and interpreted the data and were involved in drafting the manuscript, and critically revising it for important intellectual content. MM was involved in approving the final version to be published. All authors read and approved the final manuscript.

## Funding

This work was supported in part by Moonshot R&D Grant Number JPMJMS2011.

## Conflict of Interest

The authors declare that the research was conducted in the absence of any commercial or financial relationships that could be construed as a potential conflict of interest.

## Publisher's Note

All claims expressed in this article are solely those of the authors and do not necessarily represent those of their affiliated organizations, or those of the publisher, the editors and the reviewers. Any product that may be evaluated in this article, or claim that may be made by its manufacturer, is not guaranteed or endorsed by the publisher.
