# Supplementary material for: Android Robot Promotes Disclosure of Negative Narratives by Individuals With Autism Spectrum Disorders
Source: Front Psychiatry. 2022 Jun 15;13:899664. doi: 10.3389/fpsyt.2022.899664 (PMC9240260; doi:10.3389/fpsyt.2022.899664)
Supplement: Supplementary file 1 [file Data_Sheet_1.DOCX]

***Supplementary Material 1 (S1)***

One of the short exemplifications is presented in each situation. Please try to write your response in the same form as the first sentence in each numbered item.

[Script A]

1. When I was a child, I used to play outside with my friends. What about when you were a child? Please try to write your response in the same form as the preceding sentence.

In my childhood, I

2. People often say that I am a quiet person. How about you? Please try to write your response in the same form as the preceding sentence.

People often say that I

3. I live in a happy home. How about you? Please try to write your response in the same form as the preceding sentence.

I live in

4. I make mistakes from time to time, but it's not a big deal, so I try not to worry about it too much. How about you? Please try to write your response in the same form as the preceding sentence.

I make mistakes

5. My family took good care of me. How about yours? Please try to write your response in the same form as the preceding sentence.

My family

6. The situation where I feel most comfortable speaking is when I’m talking about my favorite things. How about you? Please try to write your response in the same form as the preceding sentence.

My favorite situation

7. I like to avoid conflict as much as possible and resolve the issue through discussion. How about you? Please try to write your response in the same form as the preceding sentence.

Conflict

8. I find out information I want to know by asking people and conducting research until I am satisfied. How about you? Please try to write your response in the same form as the preceding sentence.

What I want to know

9. My father is a gentle and kind man. How about your father? Please try to write your response in the same form as the preceding sentence.

My father

10. I don't like people who insult others. How about you? Please try to write your response in the same form as the preceding sentence.

Who I don't like is people who

[Script B]

1. Most of my clothes are brightly colored. How about yours? Please try to write your response in the same form as the preceding sentence.

My clothes

2. I believe that death comes to everyone. How about you? Please try to write your response in the same form as the preceding sentence.

Death

3. I sometimes think about how nice it would be if all people were kinder. How about you? Please try to write your response in the same form as the preceding sentence.

All people

4. I cannot deceive people. How about you? Please try to write your response in the same form as the preceding sentence.

What I cannot do is

5. I don’t like playing sports. How about you? Please try to write your response in the same form as the preceding sentence.

Playing sports

6. In the future, I want to be someone who can help others. How about you? Please try to write your response in the same form as the preceding sentence.

In the future

7. If I never knew my mother, I wouldn't be the person I am today. How about you? Please try to write your response in the same form as the preceding sentence.

If my mother

8. I think there's no such thing as an easy job. What do you think? Please try to write your response in the same form as the preceding sentence.

Job

9. What I secretly hope for is the happiness of those close to me. How about you? Please try to write your response in the same form as the preceding sentence.

I secretly

10. There are many things in this world that make life complicated. What do you think about it? Please try to write your response in the same form as the preceding sentence.

In this world

[Script C]

1. All my family members are cheerful people. How about your family？Please try to write your response in the same form as the preceding sentence.

My family

2. I'm sometimes disappointed with myself. How about you? Please try to write your response in the same form as the preceding sentence.

I'm sometimes

3. I love people who work hard all the time. How about you? Please try to write your response in the same form as the preceding sentence.

I love people

4. My biggest complaint is that I am busy. How about you? Please try to write your response in the same form as the preceding sentence.

My complaint is

5. I don't have any brothers or sisters, but I would have liked to have had a brother or sister. How about you? Please try to write your response in the same form as the preceding sentence.

My brother/sister

6. I learned a lot at school. How about you? Please try to write your response in the same form as the preceding sentence.

In the school

7. I think my face is very Japanese. What do you think about your face? Please try to write your response in the same form as the preceding sentence.

My face

8. I didn’t use to think much about my future, but as I grew older, I began to think about it. How about you? Please try to write your response in the same form as the preceding sentence.

Until now

9. I feel that I have more female friends than male friends. How about you? Please try to write your response in the same form as the preceding sentence.

Girl

10. What I try to remember is the excitement of success rather than my failures. How about you? Please try to write your response in the same form as the preceding sentence.

What I remember is
