# Supplementary material for: Android Robot Promotes Disclosure of Negative Narratives by Individuals With Autism Spectrum Disorders
Source: Front Psychiatry. 2022 Jun 15;13:899664. doi: 10.3389/fpsyt.2022.899664 (PMC9240260; doi:10.3389/fpsyt.2022.899664)
Supplement: Supplementary file 2 [file Data_Sheet_2.DOCX]

***Supplementary material 2 (S2)***

The order of conditions in each participant is as follows.

ID1:　P-A-H

ID2: A-P-H

ID3: H-P-A

ID4: H-A-P

ID5: P-A-H

ID6: A-P-H

ID7: H-P-A

ID8: P-A-H

ID9: A-H-P

ID10: P-H-A

ID11: H-P-A

ID12: H-A-P

ID13: A-P-H

ID14: A-P-H

ID15: P-H-A

ID16: A-H-P

ID17: P-H-A

ID18: H-P-A

ID19: A-H-P

ID20: A-H-P

ID21: H-A-P

*Notes: A=Exemplification by android robot, H=Exemplification by human interviewer, P= Exemplification being written on testing paper*
